# Novel Insights Into the Interactions Between the Gut Microbiome, Inflammasomes, and Gasdermins During Colorectal Cancer

**DOI:** 10.3389/fcimb.2021.806680

**Published:** 2022-01-17

**Authors:** Giuseppe Privitera, Nitish Rana, Franco Scaldaferri, Alessandro Armuzzi, Theresa T. Pizarro

**Affiliations:** ^1^ Department of Pathology, Case Western Reserve University School of Medicine, Cleveland, OH, United States; ^2^ Dipartimento Universitario di Medicina e Chirurgia Traslazionale, Università Cattolica del Sacro Cuore, Rome, Italy; ^3^ Centro Malattie Apparato Digerente (CEMAD), Inflammatory Bowel Disease (IBD) Unit, Unità Operativa Complessa di Medicina Interna e Gastroenterologia, Dipartimento di Scienze Mediche e Chirurgiche, Fondazione Policlinico Universitario ‘A. Gemelli’ Istituto di Ricovero e Cura a Carattere Scientifico (IRCCS), Rome, Italy; ^4^ Department of Physiology and Biophysics, Case Western Reserve University School of Medicine, Cleveland, OH, United States

**Keywords:** colitis, colorectal cancer (CRC), dysbiosis, gasdermins (GSDMs), gut microbiome, inflammasomes, intestinal mucosal immunity

## Abstract

Colorectal cancer (CRC) is one of the most prevalent and deadly forms of cancer in Western countries. Inflammation is a well-known driver of colonic carcinogenesis; however, its role in CRC extends beyond colitis-associated cancer. Over the last decades, numerous associations between intestinal dysbiosis and CRC have been identified, with more recent studies providing mechanistic evidence of a causative relationship. Nonetheless, much remains to be discovered regarding the precise implications of microbiome alterations in the pathogenesis of CRC. Research confirms the importance of a bidirectional crosstalk between the gut microbiome and the mucosal immune system in which inflammasomes, multiprotein complexes that can sense “danger signals,” serve as conduits by detecting microbial signals and activating innate immune responses, including the induction of microbicidal activities that can alter microbiome composition. Current evidence strongly supports an active role for this “inflammasome–microbiome axis” in the initiation and development of CRC. Furthermore, the gasdermin (GSDM) family of proteins, which are downstream effectors of the inflammasome that are primarily known for their role in pyroptosis, have been recently linked to CRC pathogenesis. These findings, however, do not come without controversy, as pyroptosis is reported to exert both anti- and protumorigenic functions. Furthermore, the multi-faceted interactions between GSDMs and the gut microbiome, as well as their importance in CRC, have only been superficially investigated. In this review, we summarize the existing literature supporting the importance of the inflammasome–microbiota axis, as well as the activation and function of GSDMs, to gain a better mechanistic understanding of CRC pathogenesis.

## Introduction

Colorectal cancer (CRC) represents the third most prevalent malignancy and the second cause of cancer-related deaths worldwide (Arnold et al., 2017). Aside from specific hereditary forms accounting for 3%–6% of all cases (Kanth et al., 2017), CRC is a multifactorial disorder, wherein several elements cooperatively interact in genetically susceptible hosts to promote malignant transformation (Wan et al., 2020). CRC can either arise within macroscopically normal mucosa, through the adenoma–carcinoma sequence, or as a long-term complication of chronic intestinal inflammation, such as in the case of ulcerative colitis or colonic Crohn’s disease (Brenner et al., 2014).

The gut microbiome and mucosal immune system play a critical role in the pathogenesis of CRC (Janney et al., 2020). Chronic, non-resolving inflammation has a well-established link to carcinogenesis, as some inflammatory by-products can damage DNA integrity and initiate cancer development. Furthermore, appropriate host immune responses are required for antitumor surveillance, as well as clearance of newly generated neoplastic cells. When such mechanism(s) fail, neoplastic clones can go unchecked and expand into clinically relevant malignancies. The interplay and bidirectional relationship between the gut microbiome and mucosal immune system are crucial factors in maintaining homeostasis under normal, healthy conditions; however, once this balance is disrupted, alterations in their respective functions can feed upon each other in a vicious cycle, promoting the development of intestinal pathologies, including CRC.

Compared with healthy controls, dysbiosis is consistently detected in CRC patients, with remarkable heterogeneity in microbiome composition reported across different studies (Dai et al., 2018; Wirbel et al., 2019). A major issue when considering dysbiosis is to discern between correlative and causative associations. Recent evidence supports the concept that the fecal microbiome can actively contribute to tumorigenesis. For instance, oral gavage of CRC stools increases the risk of developing high-grade dysplasia and macroscopic polyps in mice receiving azoxymethane (AOM) (Wong et al., 2017). Furthermore, the antitumorigenic effects of TNF blockade are reported to be microbiome-dependent (Yang et al., 2020). The mechanisms by which alterations in microbiome composition and/or function might result in carcinogenesis fall into three main categories. First, bacteria can exert direct genotoxicity on colonocytes. Second, shifts in microbiome function(s) and microorganism-driven metabolism can foster tumor formation. Third, the bidirectional crosstalk between the gut microbiome and immune system can promote CRC development, in that both immune and intestinal epithelial cells (IECs) can shape the intestinal microbiome, which in return, can influence the activation and functionality of the immune system. Importantly, distinction between luminal and mucosa-associated microbiomes should be made as the two can differ significantly, with speculation that these differences can functionally impact CRC pathogenesis. Luminal microbiota are more strongly implicated in metabolic exchange with the host, while mucosa-associated microbiota can directly contact and affect IECs (Chen et al., 2012). Further evidence suggests that mucosa-associated microbiome in tumors differs from that of non-neoplastic areas within the same individuals (Leung et al., 2019), and that localization of CRC (proximal vs. distal) may also influence microbiome composition (Jin et al., 2021), providing a compelling case for the importance of tumor-specific microbiome(s) in the development of CRC.

Central to the interactions between the microbiome and mucosal immune system are pattern recognition receptors (PRRs) that recognize highly conserved molecules, i.e., pathogen- and damage-associated molecular patterns (PAMPs, DAMPs). PRRs elicit innate immune responses, prompting non-specific microbicidal and proinflammatory effector functions and activating downstream adaptive immunity. Cytosolic PRRs—specifically, nucleotide-binding oligomerization domain-like receptors (NLRs)—are gaining increased attention following the discovery of the inflammasome, which can influence host–microbiome interactions, and when dysregulated, contributes to the development and progression of certain malignancies (Man, 2018).

One of the downstream effectors of inflammasome activation includes gasdermins (GSDMs), a family comprised of six structurally related proteins (GSDM A–E and DFNB59) that are primarily known for their role in pyroptosis (Broz et al., 2020). During pyroptosis, cleaved GSDM N-terminal domains assemble into oligomeric pores within the plasma membrane to initiate inflammatory cell death (Liu et al., 2021). GSDMs are expressed in a tissue-specific manner, implying different roles in the development of various physiologic and pathologic conditions, including cancer (Liu et al., 2021). Considering the well-established association between chronic intestinal inflammation and cancer (Ullman and Itzkowitz, 2011), the hypothesis that GSDM-regulated inflammation may play a role in carcinogenesis is plausible and deserves careful consideration. Furthermore, recent evidence implicates GSDM activation in non-pyroptotic functions, including differentiation, proliferation, migration, and non-lytic release of inflammatory mediators, suggesting that the contribution of GSDMs in CRC might extend beyond pyroptosis (Zou et al., 2021; Rana et al., 2022). Nonetheless, the interaction(s) between a dysregulated microbiome and GSDMs, as well as their contribution to the progression of microbiome-associated inflammation leading to CRC, is largely unknown.

The following sections will summarize current findings regarding the impact of reciprocal interactions between the gut microbiome and the inflammasome, the so-called “microbiome–inflammasome axis,” and highlight the role of downstream gasdermins toward the pathogenesis of CRC.

## Role of the Inflammasome

The inflammasome is a cytosolic multiprotein complex that senses PAMPs/DAMPs and, in response, mediates proteolytic cleavage of inflammatory proteins. Several different PRRs can contribute to inflammasome assembly, including NAIP/NLRC4 (NLR family of apoptosis inhibitory proteins/NLR family CARD domain containing-4), NLRP1 (NLR pyrin domain-containing protein-1), NLRP3, NLRP6, NLRP12, AIM2 (absent-in-melanoma-2), and PYRIN, each with its own specific activator. Each sensor can either directly bind pro-caspase-1 or recognize the adaptor protein, ASC (apoptosis-associated speck-like protein containing a CARD), which binds to pro-caspase-1 (Pandey et al., 2021). In the canonical pathway, once assembled within the inflammasome, pro-caspase-1 cleaves itself into active caspase-1, which then promotes proteolytic maturation of IL-1β and IL-18 (Dinarello et al., 2010), as well as cleavage and activation of specific pore-forming gasdermins (Broz et al., 2020).

Several studies report that inflammasome components exert protection against tumorigenesis in a mouse model of CRC, secondary to chemically induced AOM/dextran sodium sulfate (DSS) colitis. Zaki et al. demonstrate that *Nlrp3* deficiency significantly aggravates colitis and increases mortality in acute DSS-colitic mice (Zaki et al., 2010a); similarly, Allen et al. find that mice lacking *Nlrp3* are more susceptible to both acute and intermittent DSS-induced colitis and AOM/DSS-induced colitis-associated CRC (Allen et al., 2010). Both groups performed bone-marrow transplantation to assess the contribution of *Nlrp3* in hematopoietic vs. non-hematopoietic compartments. Interestingly, while the former finds that NLRP3 in non-hematopoietic cells protects against inflammation, the latter shows that *Nlrp3^−/−^* recipients of NLRP3-expressing immune cells are protected against tumorigenesis. Tissue-specific expression and function(s) of *Nlrp3* may be responsible for this contradiction. NLRP3 may exert time- and context-dependent protective effects, which may be prominent in the non-hematopoietic compartment during acute inflammation, and progressively becomes more important in hematopoietic cells during established, chronic inflammation leading to cancer.

IL-18 can stimulate epithelial regeneration and induce antitumor responses mediated by T and natural killer (NK) cells (Fabbi et al., 2015). Its administration to *Casp1*-deficient mice reduces inflammation and tumorigenesis secondary to AOM/DSS (Zaki et al., 2010b). Conversely, others show that *Il18* deletion is protective against AOM/DSS-induced colitis and CRC (Nowarski et al., 2015). This apparent dichotomy in IL-18 function may be explained by its action on soluble IL-22-binding protein. While IL-18 can directly prevent inflammation-driven carcinogenesis, it also downregulates soluble IL-22-binding protein that neutralizes IL-22, thereby indirectly enhancing the procarcinogenic effects of IL-22. IL-1β is traditionally regarded as a proinflammatory and procarcinogenic cytokine; however, both tumor-promoting and tumor-inhibiting effects are described (Baker et al., 2019). Interestingly, while the link between inflammasome function(s) and CRC pathogenesis is usually thought to be *via* inflammation, Spanlinger et al. describe a mouse model wherein tumorigenesis is uncoupled from intestinal inflammation (Spalinger et al., 2018). In this study, deletion of tyrosine phosphatase non-receptor type 2 (*Ptpn2*) in myeloid cells results in increased intestinal inflammation but fewer malignancies. Specifically, PTPN2 downregulates ASC phosphorylation, thus reducing inflammasome activity. Its absence in myeloid cells results in increased IL-1β production that, in this context, exerts anti-inflammatory, but procarcinogenic, effects. A potential explanation for these apparent contradictory roles comes from a subsequent study, showing that IL-1β can exert opposing effects on different cell types through stimulation of IL-1R, either on epithelial and T cells that promote tumorigenesis, or on myeloid cells that reduce tumor-driven inflammation (Dmitrieva-Posocco et al., 2019).

## Microbiome–Inflammasome Interplay

A role for inflammasomes in determining the biogeography of the gut microbiome has been suggested; however, research has produced inconsistent results regarding the actual relevance of various inflammasome components in shaping microbiome composition (Man, 2018). Interestingly, Galvez et al. observe that, in mice housed under specific pathobiont-free conditions, NLRP6 deficiency has an impact on microbiome composition only upon introduction of pathobionts (Gálvez et al., 2017). These findings highlight the complex relationship between inflammasomes and the gut microbiome and underscore the importance of inflammasome-mediated responses to re-establish homeostasis following dysbiotic events. Inflammasomes represent a bridge between the gut microbiome and host immune responses, with emerging evidence supporting the importance of the “inflammasome–microbiota axis” in several disease settings, including CRC (Man, 2018). For example, mice lacking *Asc* or *Nlrp6* possess dysbiotic intestinal flora and increased susceptibility to CRC, which can be transferred to co-housed wild-type controls. Notably, such susceptibility is abrogated by antibiotic treatment, reinforcing the concept that the gut microbiome plays a key role in CRC development (Hu et al., 2013). *Nlrp3^R258W^* mice, a model of cryopirin-associated periodic syndrome characterized by NLRP3 hyperactivation, display autoinflammatory disorders involving the skin, joints, and eyes, but interestingly, not the intestine. AOM/DSS-treated mice carrying this mutation are more resistant to both intestinal inflammation and CRC development, which mechanistically, is thought to occur by mutant *Nlrp3* gain-of-function processes resulting in increased IL-1β-dependent secretion of antimicrobial peptides. This confers alterations in microbiome composition and functionality, making mutant mice more resistant to intestinal diseases through a mechanism that involves expansion of T regulatory cells (Yao et al., 2017). These findings are indicative of inflammasome-dependent changes in microbiome composition and function that may, at least partially, mediate CRC pathogenesis. In addition, bacteria-derived metabolites can also influence inflammasome activity and potentially impact colonic tumorigenesis. Mice deficient in *Gpr43*—a receptor that senses short-chain fatty acids—are more susceptible to chemically induced colitis and inflammation-associated CRC. Interestingly, GPR43 engagement in colonic IECs induces NLRP3 inflammasome activation through ionic efflux (Macia et al., 2015). Another study by Levy and colleagues report that microbiota-derived taurine, histamine, and spermine modulate NLRP6-dependent release of IL-18 (Levy et al., 2015).

**Figure 1:**
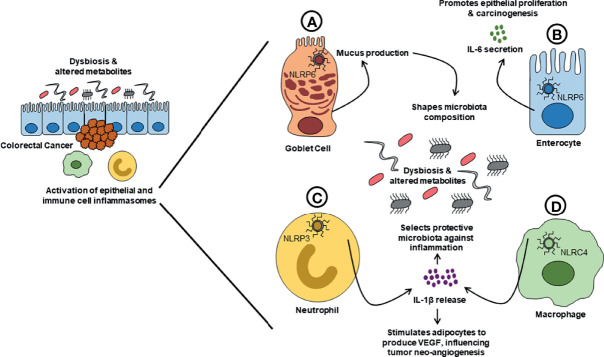
Proposed mechanisms of action for the inflammasome–microbiome axis in CRC. Alterations in composition and functionality of the gut microbiome (dysbiosis) and subsequent changes in microbial-derived metabolites can trigger inflammasome activation in both immune cells and IECs. Activation of the inflammasome(s) causes increased release of inflammatory cytokines (e.g., IL-1β and IL-18) and can lead to lytic cell death (pyroptosis). The effects of inflammasome activation can, in turn, contribute to shaping the biogeography and functions of the gut microbiome. **(A)** Upon activation of NLRP6 inflammasome, a specific population of goblet cells (GCs) communicates with other responsive GCs through GAP junctions to increase their production of mucus, which can result in quantitative and qualitative alterations of intestinal mucus that can subsequently affect microbiome composition and influence CRC development. **(B)** Activation of NLRP6 inflammasome in enterocytes induces increased production of IL-6, which stimulates IEC proliferation, and potentially promotes tumor development and growth. **(C)** Activation of NLRP3 in neutrophils induces increased secretion of IL-1β, stimulating the production of antimicrobial peptides that shapes the gut microbiome towards an anti-inflammatory phenotype, thus protecting against CRC development. **(D)** In a model of diet-induced obesity, tumor-infiltrating macrophages increase the secretion of IL-1β in response to activation of the NLRC4 inflammasome. IL-1β then stimulates adipocytes to produce VEGF, which controls tumor neoangiogenesis and influences its development. Collectively, changes in gut microbiome and activation of inflammasomes can either promote or suppress the development of CRC. CRC, colorectal cancer; IL, interleukin; NLRC, NLR family CARD domain containing; NLRP, NLR pyrin domain-containing protein; VEGF, vascular endothelial growth factor.

The impact of the inflammasome–microbiome axis on CRC might be even more subtle. Impairment in mucus production and secretion is linked to various gastrointestinal cancers, including CRC (Grondin et al., 2020). In fact, the NLRP6 inflammasome can regulate mucin-2 (MUC2) secretion from goblet cells (Wlodarska et al., 2014), and a specific subpopulation of goblet cells (i.e., sentinel goblet cells) responds to bacterial stimuli, activating the NLRP6 inflammasome, and communicates with other responsive goblet cells *via* gap junctions to produce MUC2 (Birchenough et al., 2016). In contrast, it appears that NRLP6 is not required for baseline mucus production (Volk et al., 2019). Indeed, inflammasome-dependent mucus secretion might come into play only in the presence of dysbiosis. Whether dysbiosis may contribute to CRC development through impaired mucus production is unknown, but poses a provocative hypothesis. Another interesting line of evidence comes from a model of breast cancer, wherein obesity is linked to tumor progression. Diet-induced obesity results in increased tumor burden in mice, by prompting increased NLRC4-dependent production of IL-1β from tumor-infiltrating macrophages. A direct effect of IL-1β on neoplastic cells is not observed, but rather, a proangiogenic effect mediated by adipocyte-derived vascular endothelial growth factor production (Kolb et al., 2016). While this specific mechanism has not been studied in CRC, it is interesting to consider, based on the well-accepted association of high-fat diet and obesity with colon tumorigenesis (Gaines et al., 2020). **Figure 1** summarizes the main observations to support the role of the “inflammasome–microbiota axis” in CRC.

## Inflammasome-Mediated Gasdermin Activation and Impact of the Microbiome

**Figure 2:**
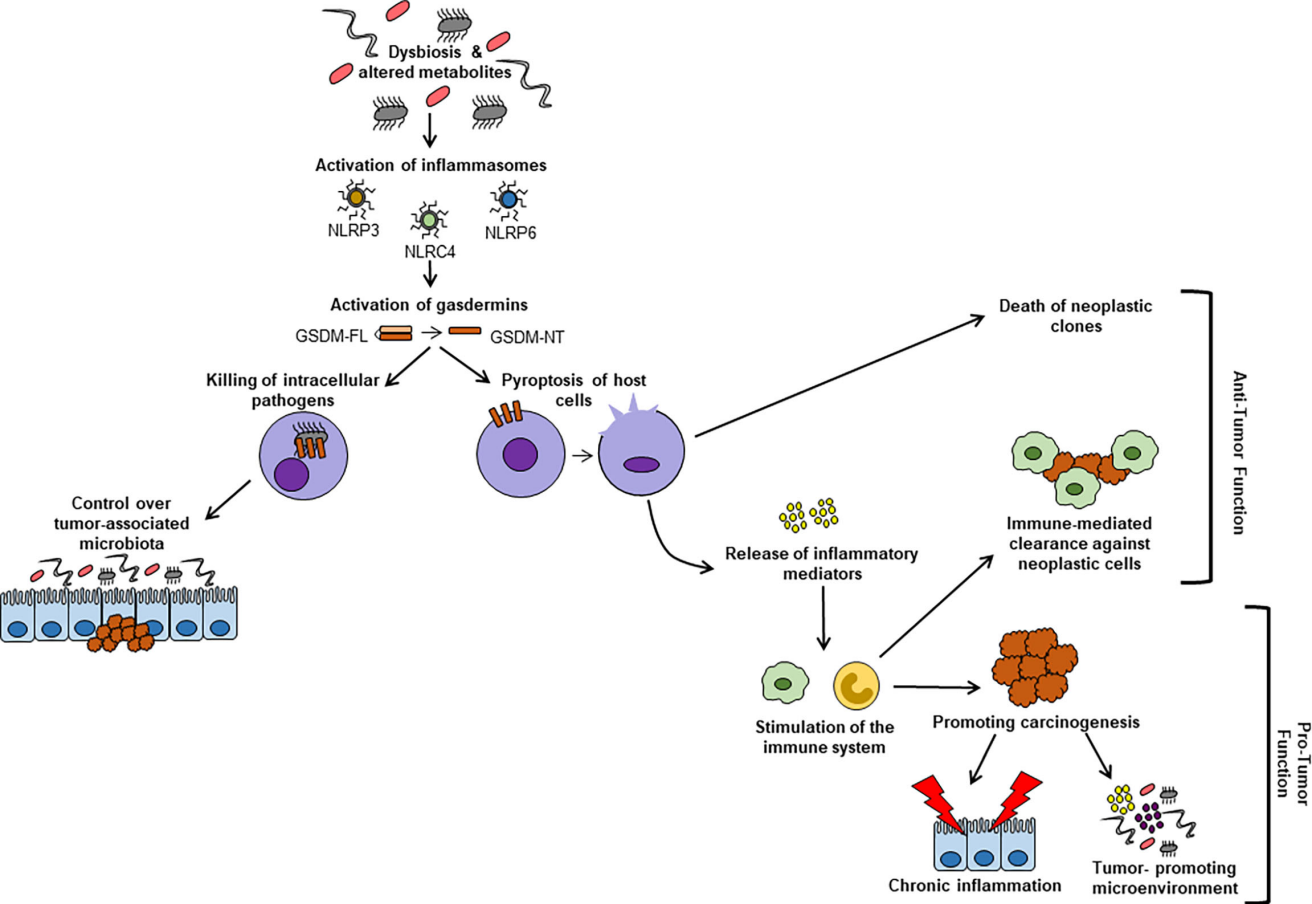
Microbiome-dependent activation of GSDMs and their contribution to CRC pathogenesis. Microbiome-derived stimuli can promote proteolytic cleavage of GSDMs into their active N-terminal domains, which assemble into pores within cell membranes and facilitate the release of inflammatory mediators, as well as pyroptotic cell death. Pyroptosis of neoplastic cells can reduce tumor burden. Inflammatory mediators released *via* pore-forming GSDMs can stimulate immune cells. This activation can result in either increased clearance of neoplastic cells or in establishing a tolerogenic, tumor-promoting microenvironment. Furthermore, GSDMB can form pores in bacterial membranes, inducing death of intracellular pathogens. *Via* this mechanism, GSDMB may also be implicated in controlling a tumor-permissive microbiome. GSDM-FL, gasdermin full-length; GSDM-NT, gasdermin N-terminal domain; NLRC, NLR family CARD domain containing; NLRP, NLR pyrin domain-containing protein.

The GSDMs are a family of structurally related proteins, which are grouped together based on their shared sequence homology and are primarily known for their execution of pyroptosis. The canonical inflammasome pathway leads to GSDM cleavage executed by caspase-1. A non-canonical pathway for GSDM activation is also described, wherein bacterial products directly activate caspases-4 and -5 to cleave GSDMs. Finally, alternative mechanisms are also present, relying on either non-inflammatory caspases (e.g., apoptotic caspases-3 or -8) or other proteases, such as granzymes and neutrophil elastases (Liu et al., 2021). The pathways of activation, as well as specific GSDMs targeted, appear to be, at least partially, dependent on the initiating stimulus and/or on the cell population involved. **Figure 2** provides an overview regarding the contribution of GSDMs to the pathogenesis of CRC.

### GSDME

*GSDME* is reduced in several cancers, including CRC, through a mechanism that involves methylation of its promoter (Yokomizo et al., 2012). GSDME protein levels can determine whether the cell, upon pro-apoptotic stimuli, is skewed toward apoptosis or pyroptosis. In “high-*GSDME*” cells, apoptotic caspase-3 cleaves GSDME and induces pyroptosis, thereby counteracting tumor growth (Rogers et al., 2017; Wang et al., 2017). Furthermore, a form of caspase-independent, GSDME-mediated cell death is described in “high-*Gsdme*” cancer cells (Zhang et al., 2020), wherein granzyme B from NK and CD8^+^ T cells can enter *via* a perforin-dependent mechanism, cleaving GSDME and activating pyroptosis, with a consequent reduction in tumor growth. The tumor-suppressing role of GSDME-mediated pyroptosis is not only related to direct killing of neoplastic cells, but also to recruitment and activation of immune cells with antitumor functions (Zhang et al., 2020). Conversely, in another study using the AOM/DSS model, GSDME-dependent pyroptosis and the subsequent release of HMGB1 are associated with colitis and tumor development (Tan et al., 2020). The possibility exists that continuous GSDME-mediated pyroptosis in non-neoplastic cells may initiate and promote tumorigenesis *via* chronic inflammation, whereas in neoplastic cells, antitumor immune responses may prevail. *GSDME* is also associated with response to anticancer therapies. The chemotherapeutic agent, lobaliplatin, acts by inducing GSDME-mediated pyroptosis in CRC cells (Yu et al., 2019), while decitabine, a DNA methylase inhibitor, increases *GSDME*, thereby boosting cell sensitivity to certain anticancer drugs (Wang et al., 2017). Finally, a role for GSDME as a potential tumor marker has also been proposed. Ibrahim et al. report that the combination of methylation of two CpG islands in *GSDME* could discriminate between normal and neoplastic tissues (Ibrahim et al., 2019).

### GSDMC

In regard to GSDMC, a role in enhancing cell proliferation during CRC is described, and its silencing can dampen tumorigenesis (Miguchi et al., 2016). GSDMC is reported to control the switch from apoptosis to pyroptosis in response to TNF. Under hypoxic conditions, PD-L1 translocates to the nucleus, where it increases *GSDMC*, which in turn, induces caspase-8-dependent pyroptosis. Interestingly, this process purportedly promotes tumorigenesis, in contrast to GSDME-induced pyroptosis (Hou et al., 2020). Another study shows that α-KG can induce GSDMC cleavage and pyroptosis in a caspase-8-dependent manner, resulting in repressed tumor growth and metastasis (Zhang et al., 2021). As such, the possibility exists that anti- or protumorigenic effects of GSDMC depend on the magnitude of pyroptosis induced. Chronic pyroptosis confined to the central region of the tumor (where hypoxic conditions prevail) may promote a tolerogenic environment and subsequent carcinogenesis (Hou et al., 2021), whereas more abrupt and diffuse pyroptosis might reduce tumor growth.

### GSDMB

The role of GSDMB in cancer remains elusive. Zhou and colleagues observe overexpression of *GSDMB* in CRC tissues. The authors describe granzyme A-induced cleavage of GSDMB in CRC cell lines, wherein granzyme A derived from either NK or CD8^+^ T cells enters the cytoplasm *via* perforin, then cleaves GSDMB that induces pyroptosis (Zhou et al., 2020). The implication of these findings is that GSDMB-mediated pyroptosis may promote CRC pathogenesis. Alternatively, a recent study implicates GSDMB-dependent non-pyroptotic functions in IEC, including proliferation, migration, and adhesion (Rana et al., 2022), which when dysregulated could also potentially lead to tumorigenesis. Of note, *GSDMB* single nucleotide polymorphisms are linked to increased susceptibility to inflammatory bowel disease (IBD) (Chao et al., 2017); as such, it is possible that GSDMB may play a role in CRC pathogenesis by promoting chronic intestinal inflammation, such as that observed in IBD.

### GSDMD

Decreased GSDMD is observed in CRC tissues, with levels negatively correlating with tumor dissemination (Ma et al., 2018), tumor aggressiveness, and survival rates after 5 years (Wu et al., 2020). In this last study, the authors observe that LPS can increase cell sensitivity to oxaliplatin in a GSDMD-dependent manner by enhancing pyroptosis. Of note, a significant increase in GSDMD is observed in inflamed colonic tissues, both in ulcerative colitis patients and in DSS colitic mice. However, whether GSDMD confers protection or promotes intestinal inflammation is a point of debate, as *Gsdmd* deficiency in DSS colitic mice is reported to be both protective and pathogenic (Bulek et al., 2020; Gao et al., 2021). The impact of GSDMD on inflammatory-driven CRC has yet to be determined and warrants further investigation.

### Influence of the Microbiome

The relationship between GSDMs and the gut microbiome is currently coming to light, although research in this area of investigation is still in its infancy. An emerging role of epithelial-derived GSDMs in host defense is gaining support; however, its potential implications for the development and progression of CRC is, at present, unclear. A recent report indicates that GSDMB is capable of forming pores specifically in bacterial membranes, while sparing host cells, thereby promoting microbicidal activity and efficient clearance of infection (Hansen et al., 2021). Similarly, a role for GSDMD-mediated pyroptosis of IECs in protecting host against intracellular oral pathogens has also been described (Luchetti et al., 2021). As most tumor-associated bacteria are localized within host cells (Nejman et al., 2020), these observations raise the tantalizing question as to whether GSDM activation in CRC might be influenced by the presence of specific bacterial strains that drive tumor-promoting functions. Finally, it is worth mentioning that a recent study demonstrates that GSDMD activation within IECs is mediated by the overgrowth of commensal flora in DSS colitic mice (Gao et al., 2021), suggesting that intestinal dysbiosis may potentially affect GSDM function(s) in inflammation-driven cancers.

## Conclusions and Future Directions

The bidirectional relationship between inflammasomes and CRC has been widely investigated, with dichotomous results reporting both pathogenic and protective functions. Since inflammasome function(s) are highly dependent on the microbiological context in which they exist, microbiome composition might dramatically influence whether inflammasome-mediated responses will be protective against, or facilitate, tumorigenesis. Unravelling the reciprocal interactions between inflammasomes and the microbiome may be critical to provide mechanistic insight into the pathogenesis of CRC.

In addition, the full impact of GSDMs and how they may influence the inflammasome–microbiome axis in the pathogenesis of CRC has yet to be determined. Interestingly, altered GSDM expression is observed in IBD (Bulek et al., 2020; Tan et al., 2020; Rana et al., 2022), in which dysbiosis is thought to exert a pathogenic role (Ni et al., 2017). Ulcerative colitis and colonic Crohn’s disease are known to increase the risk of developing CRC, especially in the event of prolonged, uncontrolled inflammation (Ullman and Itzkowitz, 2011). These observations support the hypothesis that alterations of GSDM expression and/or function(s) may be associated with dysbiosis and contribute to the development of CRC.

Chronic pyroptosis appears to be a tumor-promoting factor in colitis-associated CRC, while acute pyroptosis of neoplastic cells might reduce tumor burden. Microbial triggers can activate GSDM-mediated pyroptosis *via* the inflammasome pathway; whether microbial products can also prime granzyme-dependent pyroptosis by activating NK and T cells in the context of CRC has not yet been explored, but represents an interesting hypothesis. As different GSDMs have been implicated in CRC pathogenesis, oftentimes with opposing roles, the possibility exists that disruption of a “physiological balance” among different GSDMs may initiate and/or perpetuate the development of CRC. Further investigation is needed to unravel these intricate, and sometimes counterintuitive, interactions among the gut microbiome, inflammasomes, and GSDMs, with the end goal of novel discoveries that have a clinically meaningful impact on CRC patients.

## Author Contributions

GP researched and wrote the initial drafts. NR contributed to the written and visual content. FS and AA critically reviewed/revised the manuscript. TP conceptualized, edited, and assembled the final submitted manuscript. All authors contributed to the article and approved the submitted version.

## Funding

This work was supported by grants from the National Institutes of Health: DK091222, DK042191, and DK125293 (TP).

## Conflict of Interest

The authors declare that the research was conducted in the absence of any commercial or financial relationships that could be construed as a potential conflict of interest.

## Publisher’s Note

All claims expressed in this article are solely those of the authors and do not necessarily represent those of their affiliated organizations, or those of the publisher, the editors and the reviewers. Any product that may be evaluated in this article, or claim that may be made by its manufacturer, is not guaranteed or endorsed by the publisher.

## Abbreviations

AIM2, absent-in-melanoma-2; ASC, apoptosis-associated speck-like protein containing a CARD; AOM, azoxymethane; CRC, colorectal cancer; DAMPs, damage-associated molecular patterns; DSS, dextran sodium sulfate; GSDM, gasdermin; IBD, inflammatory bowel disease; IEC, intestinal epithelial cells; IL, interleukin; MUC2, mucin-2; NLRs, nucleotide-binding oligomerization domain-like receptors; NAIP/NLRC4, NLR family of apoptosis inhibitory proteins/NLR family CARD domain containing 4; NK, natural killer; NLRP, NLR pyrin domain-containing protein; PAMPs, pathogen-associated molecular patterns; PRRs, pattern recognition receptors; PTPN2, protein tyrosine phosphatase non-receptor type 2; VEGF, vascular endothelial growth factor.
